# Chemical Identification of Isoflavonoids from a Termite-Associated *Streptomyces* sp. RB1 and Their Neuroprotective Effects in Murine Hippocampal HT22 Cell Line

**DOI:** 10.3390/ijms19092640

**Published:** 2018-09-06

**Authors:** Seoung Rak Lee, Ji Hoon Song, Jae-Hyoung Song, Hyun-Jeong Ko, Ji Yun Baek, Tuy An Trinh, Christine Beemelmanns, Noriko Yamabe, Ki Hyun Kim

**Affiliations:** 1 School of Pharmacy, Sungkyunkwan University, Suwon 16419, Korea; davidseoungrak@gmail.com; 2 College of Medicine, University of Ulsan, Seoul 05505, Korea; jhsong.john@gmail.com; 3 College of Pharmacy, Kangwon National University, Chuncheon 24341, Korea; thdwohud@naver.com (J.-H.S.); hjko@kangwon.ac.kr (H.-J.K.); 4 College of Korean Medicine, Gachon University, Seongnam 13120, Korea; wldbsttn@naver.com (J.Y.B.); tuyantrinh@gmail.com (T.A.T.); 5 Leibniz Institute for Natural Product Research and Infection Biology e.V., Hans Knöll Institute (HKI), Beutenbergstrasse 11a, 07745 Jena, Germany; christine.Beemelmanns@hki-jena.de

**Keywords:** fungus-growing termite, *Streptomyces* sp., isoflavonoid, neuroprotective effect, glutamate

## Abstract

Insect-associated bacteria have been recognized as a very promising natural resource for discovering bioactive secondary metabolites with diverse pharmacological effects. One new isoflavonoid glycoside, termisoflavone D (**1**), together with seven known isoflavonoids (**2**–**8**), were identified from MeOH extracts of the fungus-growing termite-associated *Streptomyces* sp. RB1. The chemical structure of the new compound **1** was elucidated using comprehensive spectroscopic methods including 1D and 2D NMR, along with LC/MS analysis. The existence of two rhamnose moieties in **1** was determined with comparative NMR analysis, and the absolute configuration was elucidated using chemical reactions. The neuroprotective activities of compounds **1**–**8** were thoroughly investigated using the murine hippocampal HT22 cell line. Compound **5** prevented glutamate-induced HT22 cell death by blocking intracellular reactive oxygen species (ROS) accumulation. The present study provides the first experimental evidence for the potential use of isoflavonoids from termite-associated bacteria as lead compounds that can prevent neuronal damage induced by glutamate.

## 1. Introduction

Insects are one of the most eminently successful groups of animals and occupy almost every terrestrial environment. Because insects inhabit diverse environmental niches, pathogenic and/or symbiotic microbes have adapted to specific insects as a host system. Over millions of years of coevolution, symbiotic microbes have adapted to live in or on the insect community thereby producing biologically active metabolites important for microbial and insect survival. In turn the insect provides necessary surface space and important nutrients [1,2]. Recently, insect-associated bacteria have been recognized as a very promising natural source for discovering novel bioactive secondary metabolites [3,4,5,6]. The high rediscovery rate of known natural products has diminished the enthusiasm to explore new natural products from historically important natural sources such as medicinal plants. We have a long-standing history of investigating symbiotic bacteria associated with fungus-growing termites (*Macrotermesnatalensis*), which led to the identification of the intriguing geldanamycin analogue natalamycin A from the termite-associated *Streptomyces* sp. M56 [7]. Later, we identified macrotermycins A–D (20-membered, glycosylated, polyketide macrolactams) from a termite-associated actinomycete, *Amycolatopsis* sp. M39 [8] and termisoflavones A–C (isoflavonoids) from a termite-associated *Streptomyces* sp. RB1 [9].

In parallel ongoing studies, bacillaene A was discovered from *Bacillus* strains from *M. natalensis* colonies and showed selective inhibition of *Pseudoxylaria*, a proposed antagonistic fungus of fungus-growing termites [10]. As one example of another insect system, coprisidins A and B (naphthoquinone-oxindole alkaloids) isolated from a dung beetle-associated *Streptomyces* sp. were found to inhibit Na^+^/K^+^-ATPase [11].

**Figure 1:**
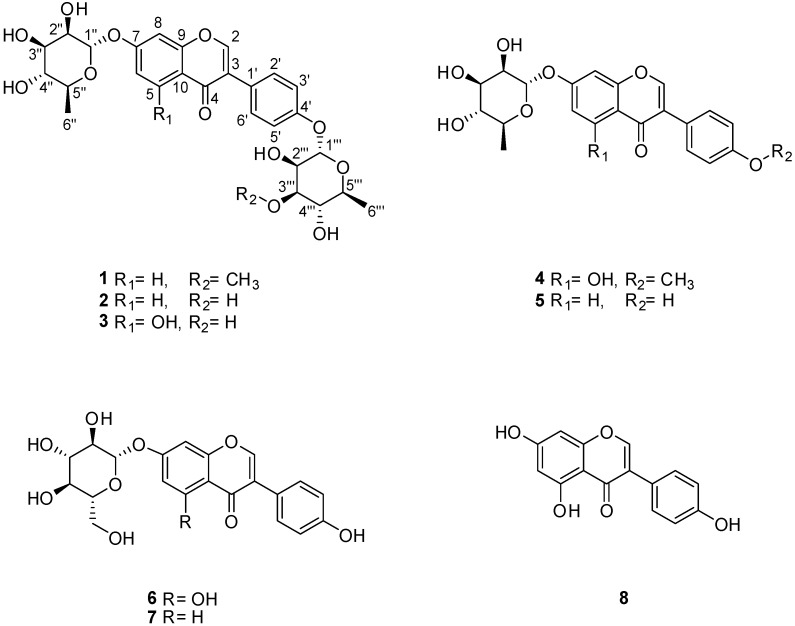
The chemical structures of compounds **1–8**.

As part of our ongoing aim to identify structurally and biologically novel compounds from termite-associated bacteria, we were recently able to identify three new isoflavonoid glycosides, termisoflavones A–C, produced by the termite-associated *Streptomyces* sp. RB1 that ameliorated cisplatin-induced kidney cell damage [9]. In our previous study of the termite-associated bacteria, we also found a renoprotectivecompound, 1-*O*-(2-aminobenzoyl)-*α*-l-rhamnopyranosidethat displayed the suppression of cisplatin-induced LLC-PK1 cells apoptosis by inhibiting the phosphorylation of c-Jun N-terminal Kinase (JNK) and p38 and cleavage of caspase-3 [12]. Inspired by the detected bioactive compounds, we continued to investigate the methanolic extracts of the termite-associated *Streptomyces* sp. RB1 by applying a comparative LC/MS- and NMR-based analysis approach. These subsequent studies resulted in the isolation of eight isoflavonoids (**1**–**8**) including a new isoflavonoid glycoside, termisoflavone D (**1**) and previously reported isoflavonoids (**4**–**7**) in our study [9] (Figure 1). Herein, we describe the LC/MS-guided isolation and structural elucidation of all the isolates (**1**–**8**) as well as the evaluation of the isolates for their neuroprotective activities.

## 2. Results and Discussion

### 2.1. LC/MS-Guided Isolation of Compounds ***1**–**8***

We used an LC/MS-guided isolation technique coupled to our house-built UV library of LC/MS to identify and separate isoflavonoids from the termite-associated *Streptomyces* sp. RB1. The high sensitivity and selectivity of the LC/MS-guided isolation method effectively reduced the analysis time and consequently enabled rapid isolation of the target compounds.

As previously reported by our group [9], several UV traces with the distinctive pattern of isoflavonoids were detected when MeOH extracts of *Streptomyces* sp. RB1 were analyzed in LC/MS. Our LC/MS-based analysis of the MeOH extract led to the isolation of eight isoflavonoids (**1**–**8**) including one new isoflavonoid glycoside (**1**) (Figure 1).

### 2.2. Structural Elucidation of the Compounds

**Figure 2:**
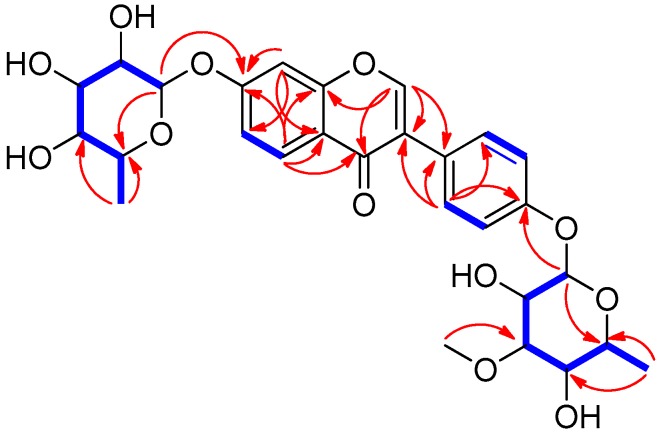
Key COSY/TOCSY (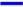) and HMBC (→) correlations for compound **1**.

Compound **1** was acquired as a yellow gum, and a molecular formula of C_28_H_32_O_12_ was deduced based on the hydrogen-adduct HR-ESIMS signal at *m*/*z* 561.1976 [M + H]^+^ (calculated for C_28_H_33_O_12_, 561.1967) (Figure S1) together with ^1^H and ^13^C NMR spectra (Table 1). The presence of hydroxyl groups and a carbonyl group was speculated from the characteristic IR absorption bands (3370 and 1681 cm^−1^, respectively). The ^1^H NMR spectrum (Figure S2) of **1** exhibited the presence of a 1,3,4-trisubstituted benzene ring [*δ*_H_7.11 (1H, dd, *J* = 9.0, 2.0 Hz, H-6), 7.19 (1H, d, *J* = 2.0 Hz, H-8), 8.08 (1H, d, *J* = 9.0 Hz, H-5)], a 1,4-disubstututed benzene ring [*δ*_H_ 7.07 (2H, d, *J* = 9.0 Hz, H-3′, H-5′), 7.42 (2H, d, *J* = 9.0 Hz, H-2′, H-6′)], and 16 proton signals responsible for two rhamnosemoieties [*δ*_H_ 1.13 (3H, d, *J* = 6.0 Hz, H-6′′′), 1.16 (3H, d, *J* = 6.0 Hz, H-6′′), 3.40 (1H, t, *J* = 9.5 Hz, H-4′′), 3.42 (1H, m, H-4′′′), 3.43 (1H, m, H-3′′′), 3.50 (1H, m, H-5′′), 3.57 (1H, m, H-5′′′), 3.76 (1H, dd, *J* = 9.5, 3.0 Hz, H-3′′), 3.96 (1H, m, H-2′′), 4.15 (1H, m, H-2′′′), 5.43 (1H, br s, H-1′′′), 5.53 (1H, br s, H-1′′)]. In particular, a distinguishable aromatic proton at *δ*_H_ 8.17 (1H, br s) in the ^1^H NMR data suggested that the core structure of **1** was an isoflavonoid moiety [9,13,14], which was also supported with comparison with our house-built UV library of LC/MS. The ^13^C NMR data showed a total of 28 carbon resonances including 15 carbons delineating the isoflavonoid scaffold as an aglycone, the 12 distinctive carbons responsible for two rhamnose moieties, and one methoxy carbon (*δ*_C_ 57.7). Detailed interpretation of the NMR spectra revealed that the structure of **1** was superimposable with termisoflavone A except that the hydroxyl group at C-5 (*δ*_C_ 159.5) of termisoflavone A was absent in **1** [*δ*_H_8.08 (1H, d, *J* = 9.0 Hz, H-5), *δ*_C_128.8 (C-5)] [9]. This deduction was unambiguously confirmed with the Heteronuclear Multiple Bond Correlation (HMBC) correlations from H-5 to C-4, C-6, C-7, and C-9, a ^3^*J*_HH_ coupling (*J* = 9.0 Hz) between H-5 and H-6 in the ^1^H NMR, and an ^1^H-^1^H correlation spectroscopy (COSY) correlation between H-5 and H-6 (Figure S3). Additionally, the aglycone of **1** was established to be daidzein based on COSY and Total Correlation Spectroscopy (TOCSY) (Figure S6) and HMBC correlations (Figure S5 and Figure 2). Two sugars, *α*-L-rhamnopyranoside and 3-*O*-methyl-*α*-L-rhamnopyranoside, were corroborated through extinctive anomeric proton signals (both H-1′′ and H-1′′′were broad singlets) and the comparison of their ^13^C NMR data with previously reported ^13^C NMR data for each sugar [9,15]. In addition, the placement of a methoxy group (*δ*_C_57.7) at C-3′′′ was verified through a ^3^*J*_CH_ correlation from the OMe (*δ*_H_3.43) to C-3′′′ (*δ*_C_82.2) in the HMBC spectrum, and ^3^*J*_CH_ correlations of H-1′′ to C-7 and H-1′′′ to C-4′ in the HMBC revealed the positions of two rhamnoses, *α*-rhamnopyranoside and 3-*O*-methyl-*α*- rhamnopyranoside, at C-7 and C-4′, respectively (Figure 2). Acid hydrolysis of **1** confirmed the daidzein as an aglycone and the identity of the two rhamnoses, *α*-L-rhamnopyranoside (([α]${}_{D}^{25}$+ 8.9, H_2_O) and 3-*O*-methyl-*α*-l-rhamnopyranoside ([α]${}_{D}^{25}$+ 9.7, H_2_O). The aglycone, daidzein, was further confirmed through LC/MS-based analysis, and the l-conformation of the two rhamnoses was confirmed through the measurement of their specific rotation values. From the collective data, the chemical structure of **1** was elucidated to be daidzein-4′-(3-*O*-methyl-*α*-rhamnopyranosyl)-7-*α*-l-rhamnopyranoside and named termisoflavone D.

**Figure 3:**
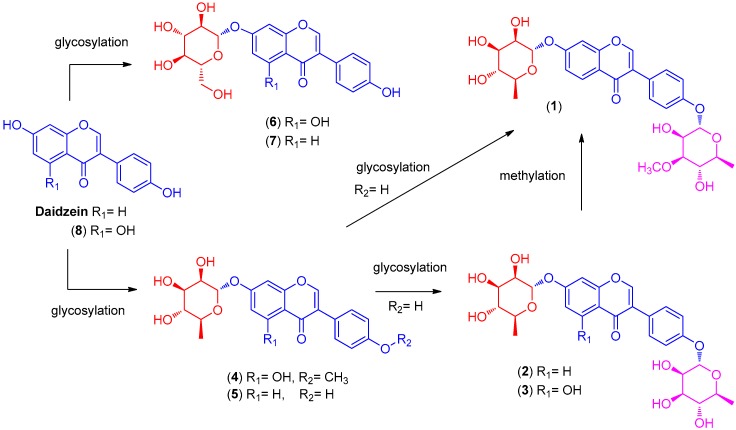
Putative sequence of transformations of compounds **1**–**8**.

The other isolated compounds were identified as daidzein-4′,7-di-*α*-l-rhamnopyranoside (**2**) [16], genistein-4′,7-di-*α*-l-rhamnopyranoside (**3**) [17], 4′-*O*-methyl-7-*O*-*α*-l-rhamnopyranosylgenistein (**4**) [18], daidzein-7-*α*-l-rhamnopyranoside (**5**) [9], genistein-7-*α*-d-glucopyranoside (**6**) [19], daidzein-7-*α*-d-glucopyranoside (**7**) [20], and genistein (**8**) [16] through comparison with previously reported spectroscopic values and LC/MS-based analysis. Among these isolates, compounds **4**–**7** had been reported in our previous study of the termite-associated *Streptomyces* sp. RB1 [9]. The identification of compounds **1**–**8** in the present study on termite-associated *Streptomyces* sp. RB1 allowed a putative sequence of transformations to be established (Figure 3).

### 2.3. Neuroprotective Activity of the Compounds Against Glutamate-Induced HT22 Cell Death

**Figure 4:**
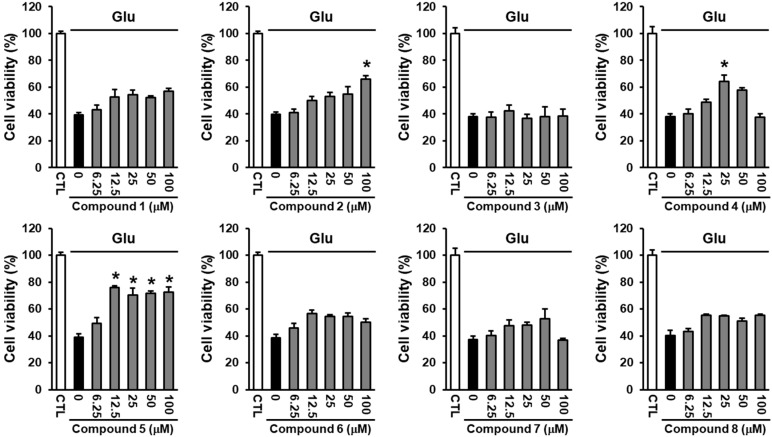
Neuroprotective effects of the isoflavonoids isolated from the termite-associated *Streptomyces* sp. RB1 in HT22 cells. The cells were treated with 5 mM glutamate and the indicated concentrations of compounds **1**–**8** (mean ± S.E.M., * *p* < 0.05 compared with glutamate-treated cells). Cell viability assays were done in triplicate for each assay and repeated at least three times.

High concentrations of glutamate, an important excitatory neurotransmitter in the brain, contribute to neuronal cell death in chronic neurodegenerative diseases and acute brain injury [21]. Several lines of research have reported that various isoflavonoids, including formononetin, genistein, and daidzein, show neuroprotective effects against ischemic stroke [22,23,24]. Therefore, we evaluated whether the isolated compounds have neuroprotective effects against glutamate-induced HT22 cell death. Treatment with glutamate reduced cell viability to 38.13 ± 2.12%, which was ameliorated by compound **5**. Among the isolated isoflavonoids, compound **5** was the most effective compound in preventing glutamate-induced HT22 cell death at low concentrations (IC_50_ = 7.19 ± 1.20 μM), while compounds **2** and **4** prevented glutamate-induced HT22 cell death at notably higher concentrations compared with compound **5** (Figure 4). The identification of eight isoflavonoids with structural differences led us to estimate a structure-activity relationship (SAR). Based on the neuroprotective effect of compound **5** observed in this study, the presence of rhamnose at C-7 may play a pivotal role in the activity, rather than glucose while the existence of rhamnose at C-4′ reduces the potency of neuroprotective effect. Although both compounds **4** and **5** have the rhamnose at C-7, compound **5** demonstrated the most significant neuroprotective activity, whereas compound **4** showed weak activity, which indicated that the daidzein aglycone can be a crucial point in exerting the neuroprotective effect. The protective mechanism of compound **5** against glutamate-induced neuronal death was further studied.

### 2.4. The Effects of Compound ***5*** on Glutamate-Induced ROS Accumulation

**Figure 5:**
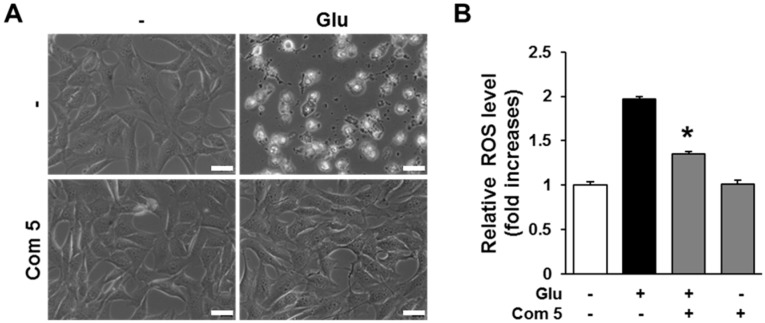
Compound **5** (Com **5**) prevented glutamate-induced HT22 cell death through inhibition of intracellular ROS accumulation. (**A**) Phase contrast microscopic images of HT22 cells were obtained after exposure to 5 mM glutamate in the presence of 12.5 μM Com **5** for 24 h. Scale bars, 20 μm. (**B**) To access the levels of intracellular ROS, HT22 cells were exposed to 5 mM glutamate in the presence of 12.5 μM Com **5** for 8 h and loaded with 2′,7′-dichlorofluorescin diacetate (H_2_DCFDA). The fluorescence intensity of dichlorofluorescin (DCF) was measured using a fluorescent microplate reader. Bars denotes the fold increase of the fluorescence intensity of DCF compared with non-treated cells (mean ± S.E.M., * *p* < 0.05 compared with glutamate-treated cells). Intracellular ROS assays were done in triplicate and repeated at least three times.

Oxidative stress is one of the most critical factors in inducing neuronal cell death in neuropathological conditions including Alzheimer’s disease, Parkinson’s disease, and ischemic stroke [25,26,27,28]. Excessive concentrations of glutamate trigger oxidative stress through an imbalance between reactive oxygen species (ROS) production and antioxidant systems [29]. Glutamate at cytotoxic concentrations induces neuronal cell death through excitotoxicity or oxidative stress. However, the blockade of intracellular ROS accumulation by *N*-acetylcysteine (NAC) and flavonoids, which are generally known to exert antioxidant properties, can prevent neuronal cell death [30,31]. This suggests that reducing the accumulation of intracellular ROS can be a potent strategy to prevent neuronal cell death. We therefore examined the effects of compound **5** against the glutamate-triggered accumulation of intracellular ROS in HT22 cells. Phase contrast microscopic images showed that the compound **5** completely blocked glutamate-induced HT22 cell death (Figure 5A). Based on phase contrast microscopic images, compound **5** completely blocked glutamate-induced HT22 cell death (Figure 5B). These data indicate that the protective effect of compound **5** is due to the blockage of intracellular ROS accumulation.

### 2.5. The Effects of Compound ***5*** on Glutamate-Induced Damage in HT22 Cells

**Figure 6:**
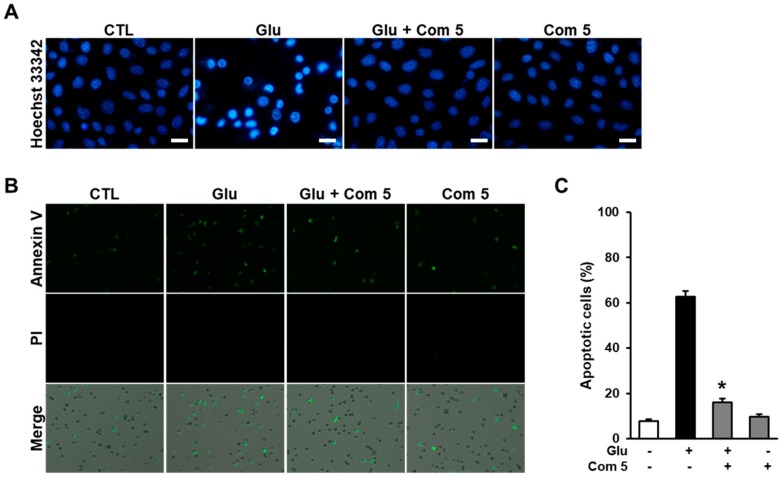
Glutamate-induced cell death was blocked by compound **5** (Com **5**) in HT22 cells. (**A**) HT22 cells were stained with Hoechst 33342 after exposure to 5 mM glutamate in the presence of 12.5 μM Com **5** for 10 h. Scale bars, 20 μm. (**B**) HT22 cells were exposed to 5 mM glutamate in the presence of 12.5 μM Com **5** for 10 h and stained with Alexa Fluor 488-conjugaged annexin V and PI. (**C**) Fluorescence images were analyzed using TaliPCApp software to determine the numbers of apoptotic cells. The bars denote the percentage of annexin V-positive (apoptotic) cells (mean ± S.E.M., * *p* < 0.05 compared with glutamate-treated cells). Image-based cytometric assays were done in triplicate and repeated at least three times.

Glutamate-induced oxidative stress can induce neuronal cell death via necrotic and apoptotic pathways. Previous studies have demonstrated that glutamate induces necrotic cell death at a relatively early time and apoptotic cell death at a late time in HT22 cells [32]. To access anti-apoptotic effect of compound **5**, we initially elucidated chromatin condensation which is one of the well-known morphological features of apoptotic cell death through staining the cells with Hoechst33342 after exposure to glutamate for 10 h. Treatment with compound **5** markedly reduced the level of chromatin condensation compared with glutamate-treated cells (Figure 6A). In addition, the cells were further labeled with Alexa Fluor 488-conjugated Annexin V and PI to determine the type of cell death. In the results, Annexin V-positive (apoptotic) cells was markedly increased by exposure to glutamate, but no PI-positive cells were detected. However, the presence of compound **5** reduced the amount of apoptotic cell death induced by glutamate (Figure 6B). Consistently, the imaging data from individual cells shows that the percentage of apoptotic cells was significantly decreased by treatment with compound **5** compared with glutamate-treated cells (Figure 6C). These results suggest that the anti-apoptotic properties of compound **5** contribute to the prevention of glutamate-induced oxidative HT22 cell death.

### 2.6. Antiviral Activities of the Compounds

Recently, some studies exploring antiviral natural products have reported that isoflavonoids exhibit potent antiviral activity by inhibiting viral protein expression and neuraminidase activity [33,34,35,36]. According to the previous studies, genistein inhibited herpes simplex virus (HSV) replication [33], and sappanone A showed potent neuraminidase (NA) inhibitory activity [34]. Moreover, calycosin-7-*O*-*β*-d-glucopyranoside displayed significant antiviral activities against CVB3 both in vitro and in vivo [36]. In the same context, we also assessed isolated compounds **1**–**8** for their antiviral activity against PR8, HRV1B, and CVB3 infection in A549, Vero, and HeLa cells, respectively. Less than 10% of the cells survived in the positive-control group (cells with virus only) after 48 h of infection. Additionally, cells treated with compounds **1**–**8** (10 μM) also had less than 10% survival (Figure S7 in Supplementary Materials). Because we could not determine any significant differences between the control and test groups, these results suggest that the compounds do not show significant antiviral activity against PR8, HRV1B, or CVB3.

## 3. Materials and Methods

### 3.1. General Experimental Procedures

Optical rotations were calculated using a Jasco P-1020 polarimeter (Jasco, Easton, MD, USA). IR spectra were obtained using a Bruker IFS-66/S FT-IR spectrometer (Bruker, Karlsruhe, Germany). Electrospray ionization (ESI) and HR-ESI mass spectra were obtained using a Waters Micromass Q-Tof Ultima ESI-TOF mass spectrometer (Waters, New York, NY, USA). NMR spectra were recorded on a Varian UNITY INOVA 800 NMR spectrometer operating at 800 MHz (^1^H) and 200 MHz (^13^C) with chemical shifts (*δ*) given in ppm. Preparative high-performance liquid chromatography (HPLC) was performed utilizing a Waters 1525 binary HPLC pump with a Waters 996 photodiode array detector (Waters Corporation, Milford, CT, USA). Semi-preparative HPLC was performed utilizing a Shimadzu Prominence HPLC system with SPD-20A/20AV UV-Vis detectors (Shimadzu, Tokyo, Japan). LC/MS analysis was conducted on an Agilent 1200 Series HPLC system (Agilent Technologies, Santa Clara, CA, USA) using an analytical Kinetex column (4.6 × 100 mm, 3.5 μm) followed by a 6130 Series ESI mass spectrometer equipped with a diode array detector. Silica gel 60 (Merck, Kenilworth, NJ, USA, 70–230 mesh and 230–400 mesh) and RP-C_18_ silica gel (Merck, 40–63 μm) were used for the column chromatography. The packing material for molecular sieve column chromatography was Sephadex LH-20 (Pharmacia, Uppsala, Sweden). Merck precoated silica gel F_254_ plates and RP-18 F_254s_ plates (Merck, Darmstadt, Germany) were used for TLC. Spots were detected on the TLC plates either under UV light or by heating after spraying with anisaldehyde-sulfuric acid.

### 3.2. Chemical Analysis of Streptomyces sp. RB1

The isolation and characterization of *Streptomyces* sp. RB1 from a *M. natalensis* nest in South Africa (Pretoria) is described in our previous study [9]. For preparative chemical analysis, *Streptomyces* sp. RB1 was grown on 60 ISP-2 agar plates (9-cm diameter) for 14 days at 30 °C. The agar was cut into squares, consolidated, and soaked overnight in MeOH. The MeOH phase was filtered and removed under reduced pressure. The crude MeOH extract (10 g) was dissolved in distilled water (250 mL) and then solvent-partitioned with EtOAc (250 mL) three times, providing 0.45 g of residue. The EtOAc-soluble fraction (0.45 g) was loaded onto a RP-C_18_ silica gel (230–400 mesh) column and fractionated using 500 mL of each solvent system of 20%, 40%, 60%, 80%, and 100% MeOH in H_2_O (fractions A–E, respectively). The fractions were then analyzed using LC/MS with a gradient solvent system of MeOH/H_2_O (1:9–1:0, flow rate 0.3 mL/min, UV 254 nm) using an analytical Kinetex column (4.6 × 100 mm, 3.5 μm). Data dereplication was performed using our house-built UV library of LC/MS. Overall, the LC/MS-guided analysis of fractions A–E indicated the presence of isoflavonoids in fractions C and D. First, fraction C (40 mg) was separated using semi-preparative reversed-phase HPLC (Phenomenex Luna C18, 250 × 10.0 mm, 5 μm, flow rate 2 mL/min) with an isocratic solvent system of 40% MeOH to yield compounds **1** (2.0 mg, *t*_R_ = 58.0 min), **2** (1.5 mg, *t*_R_ = 31.0 min), **3** (1.2 mg, *t*_R_ = 62.0 min), **4** (1.5 mg, *t*_R_ = 48.0 min), and **7** (1.0 mg, *t*_R_ = 66.5 min). Fraction D (20 mg) was separated using semi-preparative reversed-phase HPLC (Phenomenex Luna C18, 250 × 10.0 mm, 5 μm, flow rate 2 mL/min) with an isocratic solvent system of 48% MeOH to give compounds **5** (2.0 mg, *t*_R_ = 41.0 min), **6** (2.3 mg, *t*_R_ = 46.0 min), and **8** (2.5 mg, *t*_R_ = 56.0 min).

#### Termisoflavone D (**1**)

Yellow gum. [*α*]${}_{D}^{25}$− 12.5 (c 0.02, MeOH); IR (KBr) ν_max_ 3494,3370, 3204, 2966, 2306, 1681, 1619, 1409, 1054 cm^−1^; UV (MeOH) λ_max_ (log *ε*) 205 (3.90), 265 (4.12), 330 (0.45) nm; ^1^H (800 MHz) and ^13^C NMR (200 MHz) data, see Table 1; HR-ESIMS (positive ion-mode) *m*/*z* 561.1976 [M + H]^+^ (calculated as C_28_H_33_O_12_, 561.1967).

### 3.3. Acid Hydrolysis of ***1*** and Sugar Analysis

The absolute configuration of the sugar moieties was determined according to a previously published procedure [9]. Briefly, compound **1** (0.7 mg) was hydrolyzed with 1 mL of 2 N HCl for 2 h at 80 °C. The hydrolysate was solvent-partitioned with EtOAc (100 mL), and the organic solvent was evaporated *in vacuo* to afford daidzein, which was identified using LC/MS analysis and comparison with our house-built UV library of LC/MS. The aqueous layer was neutralized with an Amberlite IRA-67 column (OH^−^ form) to provide the sugar fractions. Each fraction was loaded onto a Waters C_18_ silica gel Sep-Pak Vac 6 cm^3^ column using an isocratic solvent system of MeCN-H_2_O (8:1, *v*/*v*) to give l-rhamnopyranose and 3-*O*-methyl-l-rhamnopyranose. The optical rotation values of l-rhamnopyranose and 3-*O*-methyl-*α*-l-rhamnopyranoside from **1** were determined to be [*α*]${}_{D}^{25}$+ 8.9 (c 0.02, H_2_O) and [*α*]${}_{D}^{25}$+ 9.7 (c 0.03, H_2_O), respectively.

### 3.4. Viruses, Cell Lines, and Reagents

Influenza A/PR/8 virus (PR8), human rhinovirus 1B (HRV1B), and coxsackievirus B3 (CVB3) were purchased from ATCC (American Type Culture Collection, Manassas, VA, USA). PR8, CVB3, and HRV1B were replicated in A549, Vero, and HeLa cells, respectively, at 3 °C. All cells were maintained in minimal essential medium (MEM) supplemented with 10% fetal bovine serum (FBS) and 0.01% antibiotic–antimycotic solution. Virus titers were determined using a Sulforhodamine B (SRB) assay, and the virus stock was stored at −70 °C until further use. For screening the neuroprotective activity of the samples, immortalized murine hippocampal HT22 cells were grown in Dulbecco’s modified Eagle’s medium (DMEM; Corning, Manassas, VA, USA) supplemented with 10% FBS and penicillin/streptomycin. Antibiotic–antimycotic solution, trypsin-EDTA, FBS, MEM, DMEM, and penicillin/streptomycin solution were supplied by Gibco BRL (Grand Island, NY, USA). The tissue culture plates were purchased from Falcon (BD Biosciences, Franklin Lakes, NJ, USA). SRB and glutamate (Glu) were purchased from Sigma-Aldrich (St. Louis, MO, USA). All other chemicals were of reagent grade.

### 3.5. Neuroprotective Activity Assay

To assess the neuroprotective activity of compounds **1**–**8**, HT22 murine hippocampal cells were plated onto 96-well plates and treated with 5 mM glutamate in the presence or absence of compounds **1**–**8** for 24 h. Cell viability was determined using an MTT assay kit (EZ-Cytox; Daeil Lab Service, Seoul, Korea) according to the manufacturer’s instruction. In brief, the cells were exposed to glutamate for 24 h, followed by incubation with 10 µL of EZ-Cytox reagent for 30 min. The absorbance was measured at 450 nm using an E-Max microplate reader (Molecular Devices, Sunnyvale, CA, USA).

### 3.6. Intracellular ROS Assay

The levels of intracellular ROS were determined using 2′,7′-dichlorofluorescin diacetate (H_2_DCFDA; Sigma, St. Louis, MO, USA). HT22 cells were plated on black 96-well plates at a density of 1 × 10^4^ cells per well. After exposure to 5 mM glutamate for 8 h, the cells were incubated with 10 µM H_2_DCFDA for 30 min. After washing the cells with PBS, the fluorescence intensity of DCF was measured using a microplate reader (SPARK 10M; Tecan, Männedorf, Switzerland) at 495/517 nm (ex/em).

### 3.7. Number of Apoptotic Cells

To determine the amount of apoptotic cell death, HT22 cells were seeded on 6-well plates at a density of 2 × 10^5^ cells per well and treated with 5 mM glutamate in the presence or absence of compound **5** for 10 h. The cells were first stained with 10 µM Hoechst 33342 (Sigma) to confirm chromatin condensation. Images were obtained using a fluorescent microscope (IX50; Olympus, Tokyo, Japan) equipped with a charge-coupled device (CCD) camera. Next, the cells were labeled with annexin V conjugated to Alexa Fluor 488 (Invitrogen, Eugene, OR, USA) and propidium iodide. The number of apoptotic cells was determined using a Tali Image-Based Cytometer (Invitrogen, Carlsbad, CA, USA) and analyzed with TaliPCApp software, version 1.0. The data are quantitatively represented as the percentage of Annexin V-positive cells.

### 3.8. Antiviral Activity Assay

Antiviral activity was evaluated with the SRB method using cytopathic effect (CPE) reduction, as previously reported [37]. To screen compounds **1**–**8**, A549, Vero, and Hela cells (2 × 10^4^ cells/well) were seeded in each well of a 96-well plate and incubated with 10 μM individual compounds. The cells were incubated for 2 days until the target CPE was achieved. After incubation in ice-cold 70% acetone for 30 min, the cells were stained with 0.4% (*w*/*v*) SRB in 1% acetic acid solution. Bound SRB was solubilized with 10 mM unbuffered Tris base solution, and the absorbance was measured at 562 nm using a VersaMax microplate reader (Molecular Devices, Palo Alto, CA, USA) with a reference absorbance of 620 nm. The percentage of viable cells was calculated, as previously reported in study [37].

### 3.9. Statistical Analysis

All data described in this study were repeated at least three times and are represented as the mean ± S.E.M. Statistical significance was determined by the one-way analysis of variance (ANOVA). Data were considered statistically significant at *p*-values less than 0.05.

## 4. Conclusions

Insect-associated microbes are a prolific source of structurally novel natural products with many important biological and pharmacological activities [5,38,39,40,41]. In our ongoing study of termite-associated bacteria, investigation of the MeOH extract of the fungus-growing termite-associated *Streptomyces* sp. RB1 using LC/MS-guided isolation techniques for isoflavonoids led to the isolation and identification of one new isoflavonoid glycoside, termisoflavone D (**1**), along with seven known isoflavonoids (**2–8**). Although none of the isolated compounds had antiviral effects, daidzein-7-*α*-l-rhamnopyranoside (**5**) showed a strong protective effect on glutamate-induced cell death in HT22 cells by diminishing the accumulation of intracellular ROS. Our experimental results provide the possibility of insect-associated microbes as an interesting research topic for new bioactive compounds.

## Figures and Tables

**Table 1:** ^1^H (800 MHz) and ^13^C (200 MHz) NMR data of **1** in CD_3_OD. ^a^

Position	1	
	*δ*_C_^b^	*δ*_H_ (*J* in Hz)
2	155.6 d	8.17 br s
3	126.2 s	
4	178.3 s	
5	128.8 d	8.08 d (9.0)
6	117.4 d	7.11 dd (9.0, 2.0)
7	162.8 s	
8	104.9 d	7.19 d (2.0)
9	159.7 s	
10	120.3 s	
1′	127.4 s	
2′, 6′	131.8 d	7.42 d (9.0)
3′, 5′	117.8 d	7.07 d (9.0)
4′	158.2 s	
1”	100.3 d	5.53 br s
2”	72.0 d	3.96 m
3”	72.4 d	3.76 dd (9.5, 3.0)
4”	73.8 d	3.40 t (9.5)
5”	71.7 d	3.50 m
6”	18.4 q	1.16 d (6.0)
1′′′	99.9 d	5.43 br s
2′′′	68.3 d	4.15 m
3′′′	82.2 d	3.43 m
4′′′	72.9 d	3.42 m
5′′′	71.0 d	3.57 m
6′′′	18.4 q	1.13 d (6.0)
3′′′-OMe	57.7 q	3.43 s

^a^ Coupling constants (in parentheses) are in Hz. ^b 13^C NMR data were assigned based on HSQC (Figure S4) and HMBC experiments.

## Supplementary Materials

1D NMR, 2D NMR, and HRESIMS of **1** and antiviral activity of compounds **1**–**8** are available free of charge on the Internet.
