# Supplementary material for: Chemical Identification of Isoflavonoids from a Termite-Associated Streptomyces sp. RB1 and Their Neuroprotective Effects in Murine Hippocampal HT22 Cell Line
Source: Int J Mol Sci. 2018 Sep 6;19(9):2640. doi: 10.3390/ijms19092640 (PMC6164413; doi:10.3390/ijms19092640)
Supplement: Supplementary file 1 [file ijms-19-02640-s001.pdf]

International Journal of
Molecular Sciences

MDPI

Supplementary Information

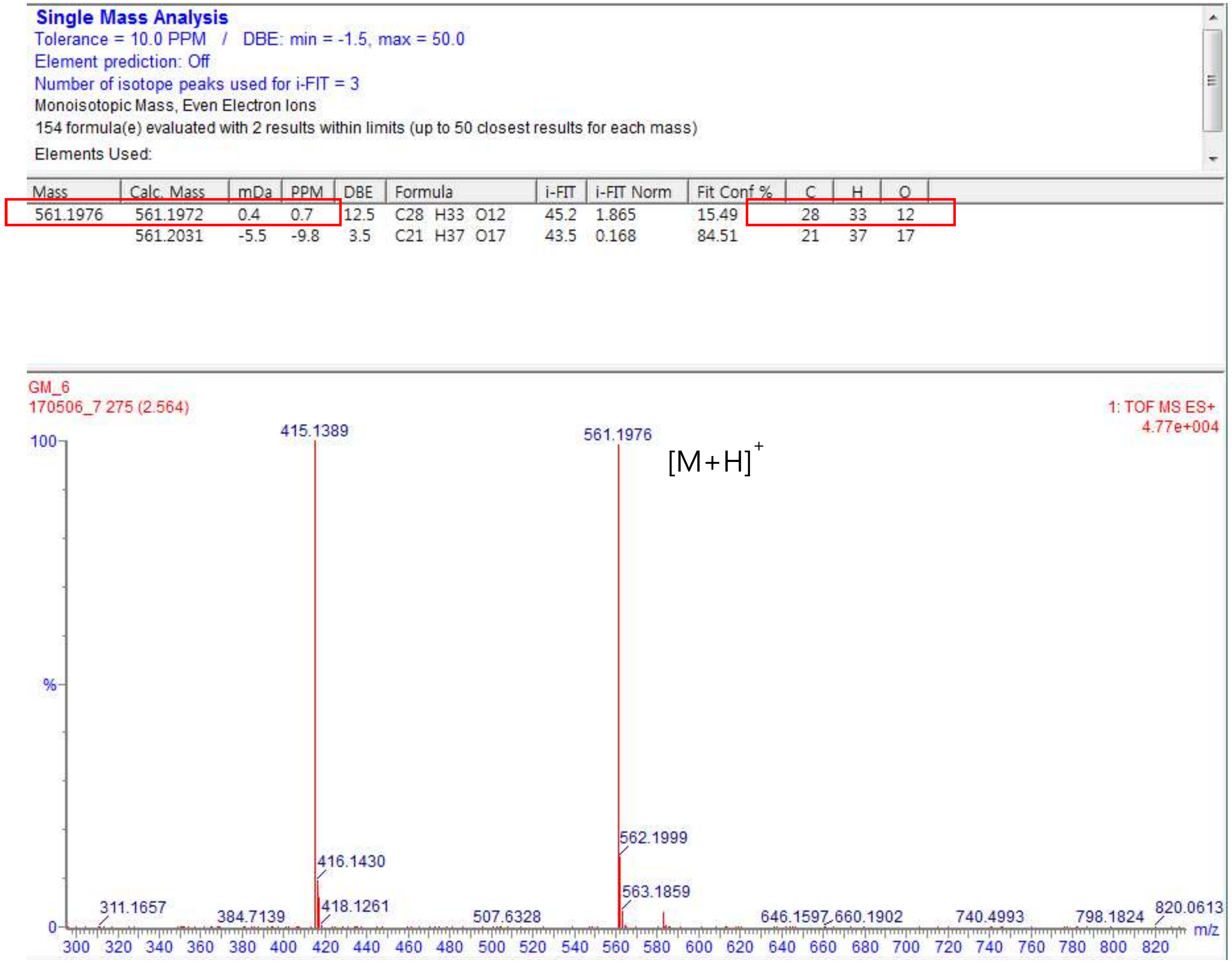

Figure S1. The HR-ESIMS data of **1.**

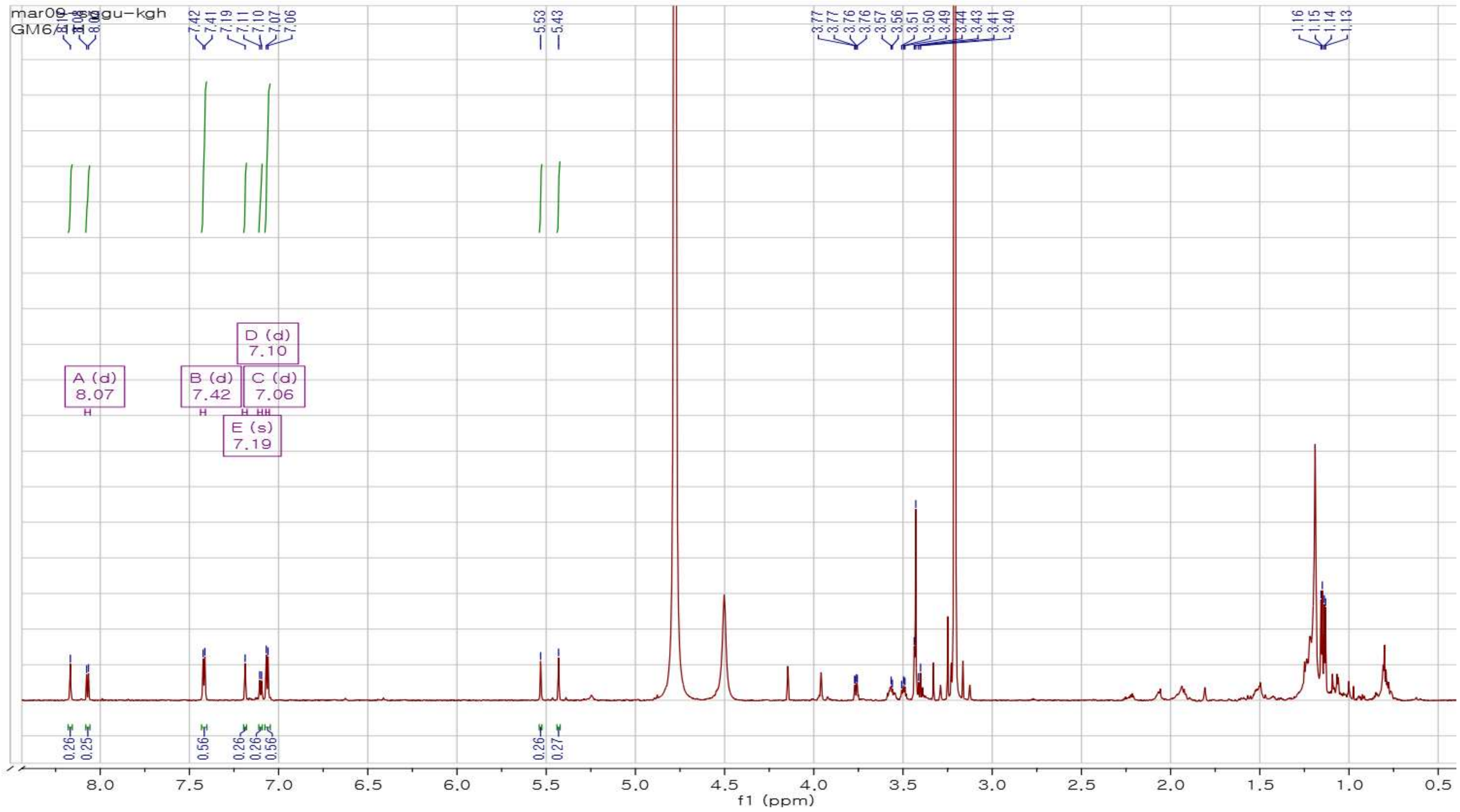

Figure S2. The ^{1}H NMR spectrum of **1** (CD_3OD, 800 MHz).

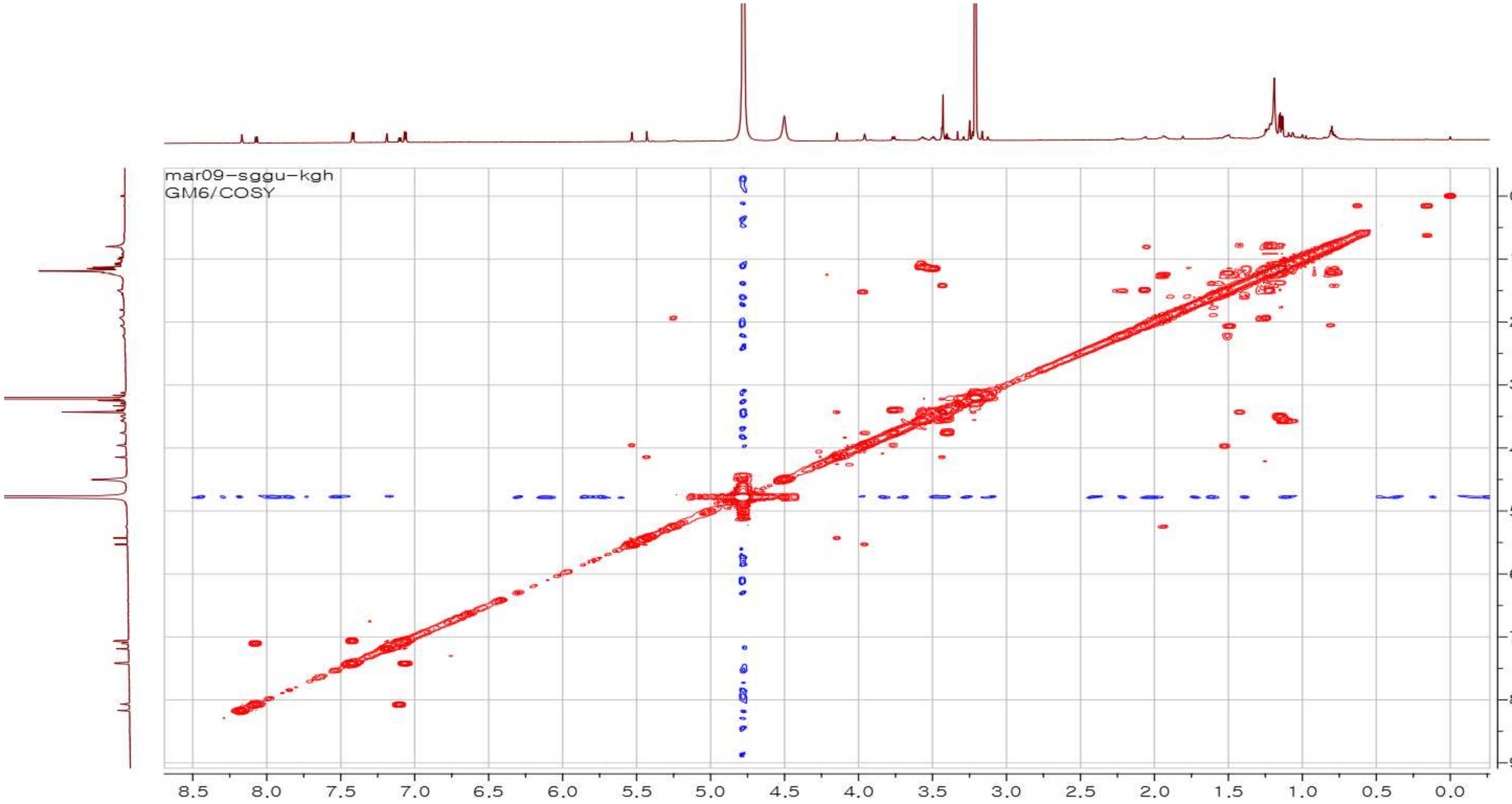

Figure S3. The ^{1}H-^{1}H COSY spectrum of **1** (CD_3OD).

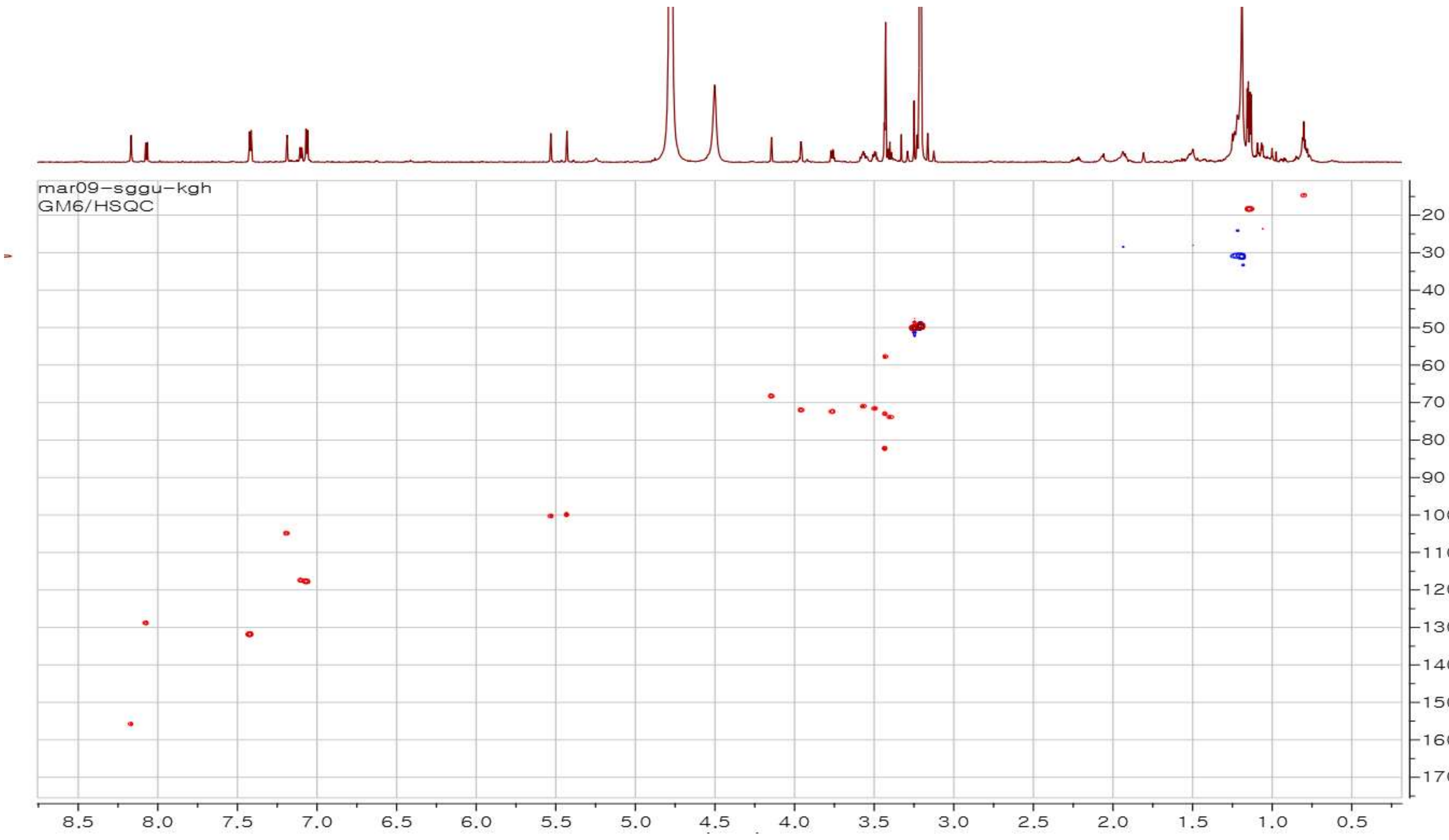

Figure S4. The HSQC spectrum of **1** (CD_3OD).

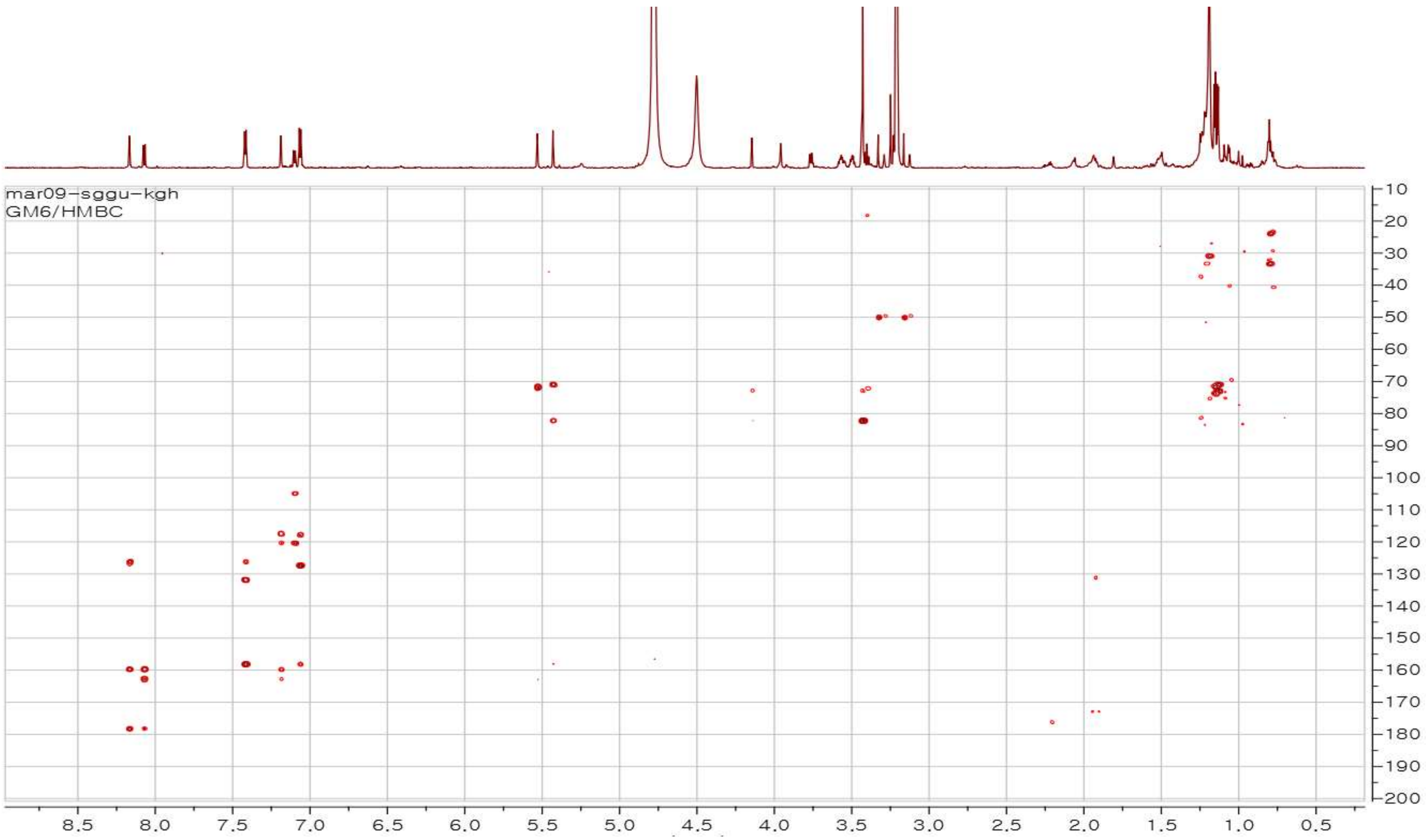

Figure S5. The HMBC spectrum of **1** (CD_3OD).

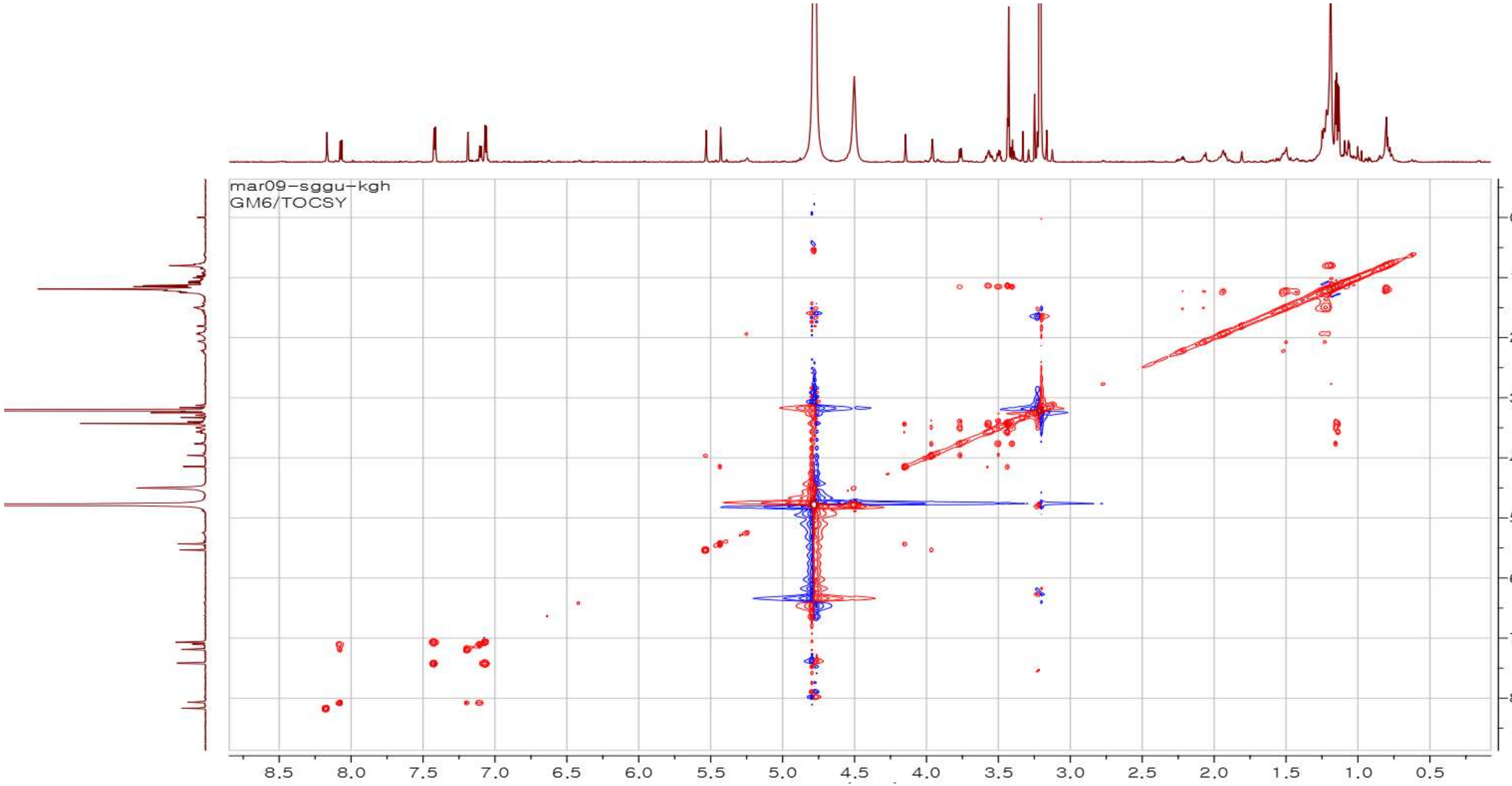

Figure S6. The TOCSY spectrum of **1** (CD_3OD).

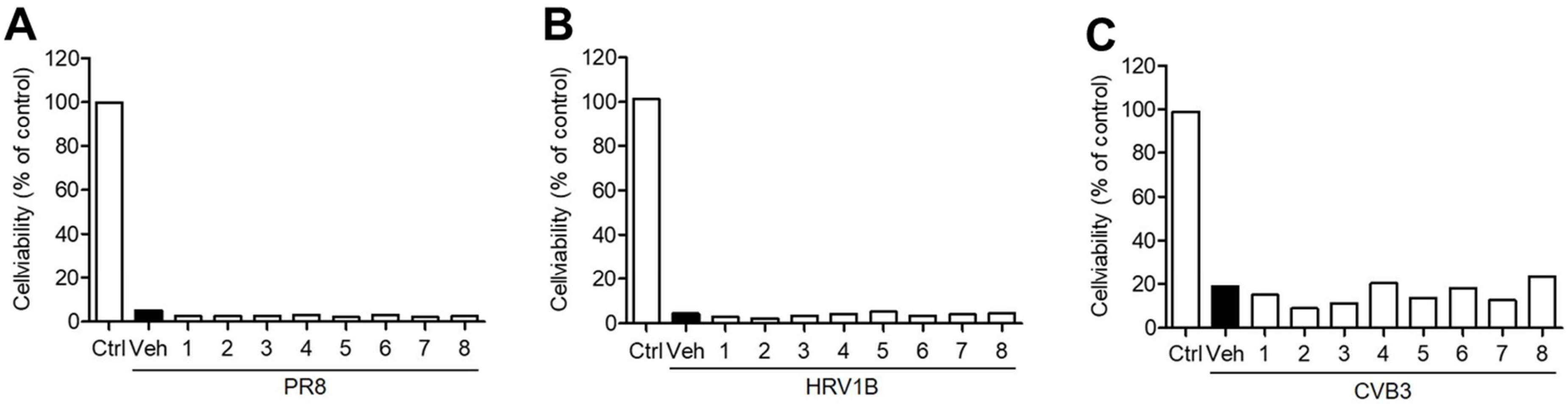

Figure S7. The antiviral activity of compounds 1-8 against PR8, HRV1B, and CVB3 in vitro. In A549, Vero and Hela cells, the antiviral activity of compounds **1-8** were evaluated based on its effects on cell viability after infection with PR8 (A), HRV1B (B), CVB3 (C). Cell viability (%) was calculated based on results of the SRB assay.
